# The utility of delta neutrophil index in differentiation of pulmonary tuberculosis from community acquired pneumonia

**DOI:** 10.1038/s41598-018-30967-9

**Published:** 2018-08-17

**Authors:** Byung Woo Jhun, Yun Su Sim, Tae Rim Shin, Dong-Gyu Kim

**Affiliations:** 1 Division of Pulmonary, Allergy and Critical Care Medicine, Department of Internal Medicine, Hallym University Kangnam Sacred Heart Hospital, Seoul, South Korea; 2 Division of Pulmonary and Critical Care Medicine, Department of Medicine, Samsung Medical Center, Sungkyunkwan University School of Medicine, Seoul, South Korea

## Abstract

No data exist on the usefulness of the delta neutrophil index (DNI) to discriminate pulmonary tuberculosis (PTB) from community-acquired pneumonia (CAP). We performed a retrospective cohort study involving patients with PTB (n = 62) and CAP (n = 215), and compared their initial DNI levels. The median DNI values were 0% (interquartile ranges [IQR] 0–0.2%) and 1.6% (IQR 0.7–2.9%) in PTB and CAP, respectively, which was significantly lower in PTB patients (*P* < 0.001). Sixty-nine percent of patients with PTB had DNI value of 0%; however, only 15% of patients with CAP had 0% DNI. The discriminatory power of the DNI for diagnosing PTB was high with 89% sensitivity and 67% specificity at a DNI cut-off ≤ 1.0% (area under the curve, 0.852). The diagnostic sensitivity and negative predictive value (NPV) for PTB were 89% (55/62) and 95% (145/152) at the DNI cut-off ≤ 1.0%, respectively, and in multivariate analyses after adjusting for other factors (smoking, no fever, upper lobe involvement), DNI ≤ 1.0% remained significant (odds ratio, 15.265; *P* < 0.001). We demonstrated that the DNI was lower in PTB compared with CAP, and an initially elevated DNI (>1.0%) may be useful to rule out the possibility of PTB due to its high NPV.

## Introduction

*Mycobacterium tuberculosis* is an acid-fast bacillus that can cause pulmonary and extra-pulmonary tuberculosis^1,2^. Tuberculosis is a communicable infectious disease with high morbidity and mortality, which remains a major public health burden worldwide. According to the 2016 World Health Organization global report, tuberculosis was one of the top 10 causes of death worldwide, ranking above acquired immune deficiency syndrome, and there were an estimated 10.4 million newly diagnosed tuberculosis cases worldwide^3^.

One of the most concerning issues faced by physicians in tuberculosis-endemic areas is the difficulty discriminating between pulmonary tuberculosis (PTB) and community-acquired pneumonia (CAP) caused by non-mycobacterial agents^4^. Because the clinical features of PTB are nonspecific or similar with those of CAP in many cases^5,6^ and the diagnostic sensitivity of a microscopic examination for diagnosing tuberculosis is only about 60%, it is difficult to accurately discriminate PTB from CAP in the early stages of the diagnostic process, particularly when managing patients with mild symptoms. Moreover, initially inappropriate antibiotic therapy for PTB, particularly newer fluoroquinolones commonly prescribed for CAP, may delay the diagnosis of PTB and can be associated with poor prognosis or drug-resistant PTB^7–9^. Thus, identifying a marker to discriminate PTB from CAP early is important; however, insufficient data for this are available, despite the fact that some studies have suggested several markers, such as C-reactive protein (CRP), procalcitonin, soluble triggering receptor, and the neutrophil-lymphocyte ratio, as discriminatory markers^10–13^.

The delta neutrophil index (DNI) was developed to measure the fraction of immature granulocytes more reliably and rapidly using an automatic hematologic analyzer ADVIA2120 (Siemens Healthcare Diagnostics) and the myeloperoxidase (MPO) and nuclear lobularity channels^14,15^. Data have shown that the DNI could be useful for distinguishing between bacterial and viral meningitis^16^, kidney rejection and pyelonephritis^17^, and CAP and upper respiratory infection^18^. However, no data are available regarding the clinical usefulness of the DNI for discriminating PTB from CAP. Therefore, in this study, we compared initial DNI levels between patients with PTB and CAP and evaluated how well the DNI value predicted PTB compared with other commonly used inflammatory blood markers.

## Methods

### Study subjects

We retrospectively reviewed the medical records of consecutive adult patients admitted with respiratory disease between November 2015 and December 2016 at Hallym University Kangnam Sacred Heart Hospital in Seoul, South Korea. In total, 422 cases of PTB and CAP were identified. Of these, intensive care unit admission cases due to severe respiratory failure or shock (n = 122), referred cases already receiving therapy (n = 20), and readmission cases (n = 4) were excluded. Finally, 277 patients with PTB (n = 62) and CAP (n = 215) who were admitted to the general ward were included in the analysis. The Institutional Review Board of the Hallym University Kangnam Sacred Heart Hospital approved this study and permitted the review and publication of patient records. The requirement for informed consent by individual patients was waived due to the retrospective design of the study.

The diagnosis of PTB was considered definitive when: (1) microbiological examination (acid-fast bacillus stain, polymerase chain reaction, or acid-fast bacillus culture) using respiratory tract specimens confirmed infection with *M. tuberculosis*^3,19^; or (2) the patient’s clinical presentation met the criteria for clinical active PTB established by the World Health Organization, when microbiological tests for tuberculosis were negative^20^. Tuberculosis pleurisy was defined when the lymphocytic exudate with pleural fluid adenosine deaminase level was ≥40 U/L and clinical improvement was observed after anti-tuberculosis treatment. CAP was defined as the presence of a new infiltrate on chest radiography without evidence of PTB plus at least one of the following: (1) respiratory symptoms including cough, sputum, dyspnea, or pleuritic chest pain; (2) fever (body temperature ≥38.0 °C) or hypothermia (body temperature <35.0 °C); or (3) abnormal breath sounds on auscultation^21^.

### Delta neutrophil index measurement

The DNI value was automatically calculated by cell analyzer, as previous studies described^14,15,22,23^. The DNI value was measured in peripheral blood using an automatic cell analyzer (ADVIA 2120 Hematology System, Siemens Healthcare Diagnostics, Forchheim, Germany). After red blood cell lysis, cell size and stain intensity were measured by the tungsten-halogen-based optical system of the MPO channel to count and differentiate granulocytes, lymphocytes, and monocytes based on their size and MPO content. This was followed by cell counting and classification according to size, lobularity, and nuclear density, using the laser diode-based optical system of the lobularity nuclear density channel counted. The DNI value was calculated by the following formula and presented as a percentage (%): DNI = [neutrophil and the eosinophil subfractions measured in the MPO channel by a cytochemical MPO reaction] − [the polymorphonuclear neutrophil subfraction measured in the nuclear lobularity channel by the reflected light beam]^17^.

### Patient management and data collection

The microbiological etiologies of all patients suspected of having PTB or CAP were evaluated in peripheral blood, sputum, bronchoalveolar fluid, nasopharyngeal or oropharyngeal specimens, or pleural fluid using the following tests; microbiological culture for bacteria; a multiplex real-time polymerase chain reaction (PCR) test for bacterial agents including *Streptococcus pneumoniae*, *Haemophilus influenzae*, *Mycoplasma pneumoniae*, and *Chlamydia pneumoniae* etc.; a multiplex real-time PCR for common respiratory viruses; and a urinary antigen test for *Streptococcus pneumoniae* or *Legionella pneumophila*.

The DNI value was routinely included in complete blood count tests at our institution, thus, initial DNI values before starting treatment for PTB and CAP were available for all patients. All data for clinical characteristics including age, gender, smoking history or underlying disease, laboratory examinations including the DNI, white blood cell (WBC) count, and C-reactive protein (CRP), and radiographic findings at the time of initial admission were collected anonymously.

### Statistical analysis

Data on categorical variables are presented as numbers and percentages, and data on continuous variables are presented as medians and interquartile ranges (IQRs). Data were compared using a chi-square or Fisher’s exact test for categorical variables and by the Mann–Whitney U test for continuous variables. Sensitivity, specificity, positive predictive value (PPV), and negative predictive value (NPV) of the DNI for diagnosing PTB were calculated using standard definitions. The predictive value of inflammatory markers, including the DNI for PTB, was evaluated by receiver operating characteristic (ROC) curve analysis, and the area under the curve (AUC) was also calculated. Multivariate analyses with logistic regression were performed to identify factors to predict PTB. Statistical analyses were performed using PASW software (ver. 22.0; SPSS Inc., Chicago, IL, USA) and a two-sided *P*-value < 0.05 was considered to indicate statistical significance.

### Ethics approval and consent to participate

The Institutional Review Board of the Hallym University Kangnam Sacred Heart Hospital approved this study and permitted the review and publication of patient records.

## Results

### Clinical characteristics of patients with PTB and CAP

In total, 62 patients with PTB and 215 patients with CAP were included in the analysis, and all were non-immunocompromised based on negative human immunodeficiency virus infection test results. Clinical characteristics of PTB patients and CAP patients are summarized in Table 1. Median ages were 52 years (IQR, 37–71 years) and 60 years (IQR, 42–75 years) in patients with PTB and CAP, respectively, and more than half of patients were male in both groups. The rate of never smokers was higher in the CAP group (*P* = 0.026); no differences in age, gender, or underlying pulmonary or extra-pulmonary disease were observed among the two groups, except history of PTB treatment (16% in PTB group vs. 7% in CAP group, *P* = 0.048). All patients had respiratory symptoms or signs; rates of cough (*P* = 0.005), sputum (*P* < 0.001), and fever (*P* < 0.001) were significantly higher in CAP group, and hemoptysis (*P* = 0.007) was more frequently observed in PTB group. No significant differences in the rate of dyspnea or severe disease (pneumonia severity index ≥ class III) were observed among the two groups. Twenty-seven (44%) of the 62 patients with PTB had positive results on the sputum acid-fast bacillus stain test.

**Table 1:** Comparisons of clinical characteristics of patients with PTB and CAP.

Characteristics	PTB (n = 62)	CAP (n = 215)	*P*-value
Age (year)	52 (37–71)	60 (42–75)	0.068
Gender (male)	39 (63)	111 (52)	0.148
Never smoker	38 (61)	153 (71)	0.026
Underlying pulmonary disease			
Chronic obstructive pulmonary disease	2 (3)	23 (11)	0.080
Asthma	—	11 (5)	NA
Previous PTB	10 (16)	16 (7)	0.048
Bronchiectasis	2 (3)	7 (3)	0.999
Lung cancer	1 (2)	5 (2.3)	0.999
Underlying extra-pulmonary disease			
Heart failure/angina	4 (7)	23 (11)	0.466
Hypertension	11 (18)	56 (26)	0.238
Diabetes mellitus	11 (18)	24 (11)	0.193
Systemic lupus erythematosus	1 (2)	—	NA
Cerebrovascular accident	2 (3)	8 (4)	0.999
Viral hepatitis	—	2 (1)	NA
Chronic alcoholics	2 (3)	2 (1)	0.217
Presenting symptoms or signs			
Cough	34 (55)	159 (74)	0.005
Sputum	14 (23)	129 (60)	<0.001
Fever (≥38.3 °C)	8 (13)	117 (54)	<0.001
Hemoptysis	5 (8)	2 (1)	0.007
Dyspnea (≥mMRC scale II)	12 (19)	49 (23)	0.607
Pneumonia severity index ≥ class III	23 (37)	73 (34)	0.652
Sputum AFB smear positive	27 (44)	—	NA

Data are shown as median (interquartile range) or number (%). *AFB* acid fast bacillus; *CAP* community acquired pneumonia; *mMRC* modified medical research council; *PTB* pulmonary tuberculosis; *NA* not applicable; *–* negative.

### Laboratory findings of patients with PTB and CAP

**Figure 1:**
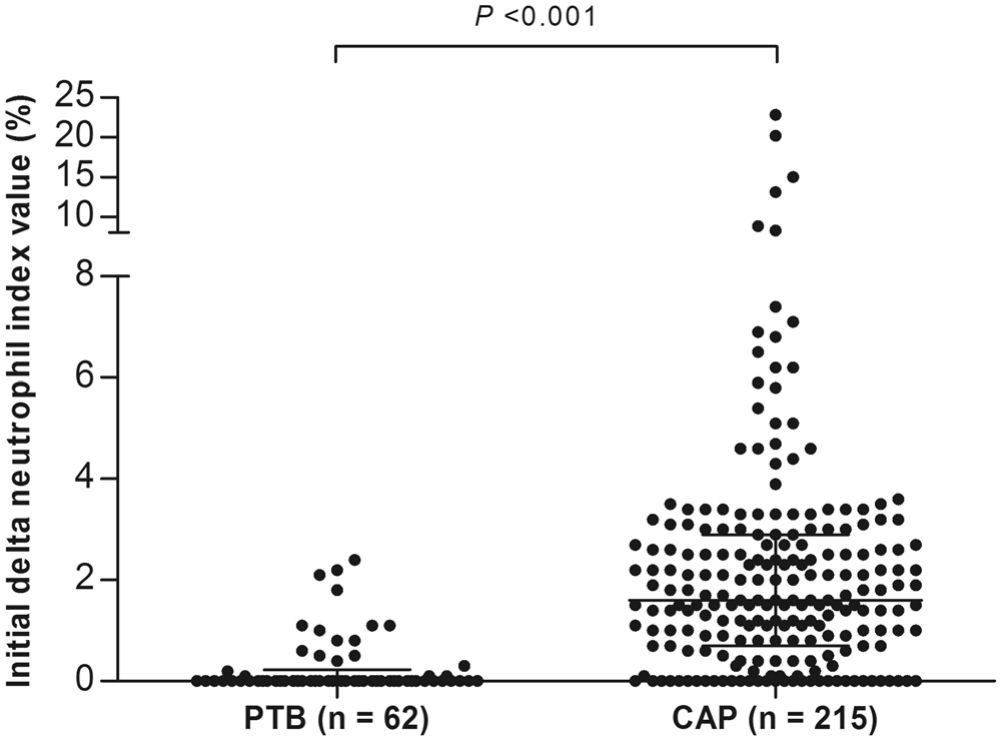
Comparisons of delta neutrophil index between patients with pulmonary tuberculosis and community acquired pneumonia. PTB, pulmonary tuberculosis; CAP, community acquired pneumonia.

We compared initial laboratory findings including WBC, DNI, and CRP levels between patients with PTB and CAP (Table 2). In patients with PTB, the median WBC count, the DNI, and CRP were 6625/μL (IQR, 5528–9065/μL), 0% (IQR, 0–0.2%), and 21.7 mg/L (IQR, 4.2–75.6 mg/L), respectively, which were all significantly lower than those of patients with CAP (all *P* < 0.001 for WBC, DNI, and CRP values). Initial DNI values in patients with PTB ranged from 0–2.4% and 69% (43/62) of patients with PTB initially had a DNI value of 0%. In contrast, initial DNI values in patients with CAP ranged from 0–22.8%, which was significantly higher than that of patients with PTB, and only 15% (32/215) of patients with CAP had an initial DNI value of 0% (Fig. 1).

**Table 2:** Comparisons of initial laboratory findings between patients with PTB and CAP.

Characteristics	PTB (n = 62)	CAP (n = 215)	*P*-value
Inflammatory markers			
WBC count (/μL)	6625 (5528–9065)	9190 (6540–13400)	<0.001
DNI (%)	0 (0–0.2)	1.6 (0.7–2.9)	<0.001
CRP (mg/L)	21.7 (4.2–75.6)	81.3 (34.8–133.7)	<0.001
Microbiological results			
*Mycobacterium tuberculosis*	46 (74)*	—	NA
Non-mycobacterial agents	10 (16)	144 (67)	<0.001
*S. pneumoniae*	3/10	25/144	
*H. influenzae*	2/10	17/144	
*M. pneumoniae*	—	4/144	
*S. pneumoniae* + *H. influenzae*	5/10	47/144	
*S. pneumoniae* + *M. pneumoniae*	—	15/144	
*H. influenza* + *M. pneumoniae*	—	2/144	
*S. pneumoniae* + *H. influenza* + *M. pneumoniae*	—	17/144	
*K. pneumoniae*	—	7/144	
*P. aeruginosa*	—	3/144	
*S. aureus*	—	3/144	
*C. pneumoniae*	—	1/144	
*M. catarrhalis*	—	1/144	
*B. pertussis*	—	1/144	
*L. pneumophila*	—	1/144	
Viral agents	3/48 (6)	35/130 (27)	
Rhinovirus	1/3	9/35	
Metapneumovirus	—	9/35	
Influenza A or B	2/3	7/35	
Parainfluenza virus	—	3/35	
Coronavirus	—	3/35	
Respiratory syncytial virus	—	3/35	
Influenza A + Respiratory syncytial virus	—	1/35	

Data are shown as median (interquartile range) or number (%). *CAP* community acquired pneumonia; *CRP* C-reactive protein; *DNI* delta neutrophil index; *PTB* pulmonary tuberculosis; *WBC* white blood cell count; not applicable; *–* negative. *Twelve patients were diagnosed with tuberculous pleurisy with (10/12) or without (2/12) suspected pulmonary lesion and 4 patients were compatible with clinical active PTB.

Non-mycobacterial agents and viral agents were identified in 144 (67%) and 35 (35/130, 27%) patients with CAP, respectively, which were both significantly higher isolation rates compared with those of patients with PTB. However, 10 patients with PTB had co-infections with non-mycobacterial agents, such as *S. pneumoniae* or *H. influenza*, and three PTB patients had viral etiologies, such as rhinovirus or influenza virus.

### ROC curve analysis of inflammatory markers for diagnosing PTB

**Figure 2:**
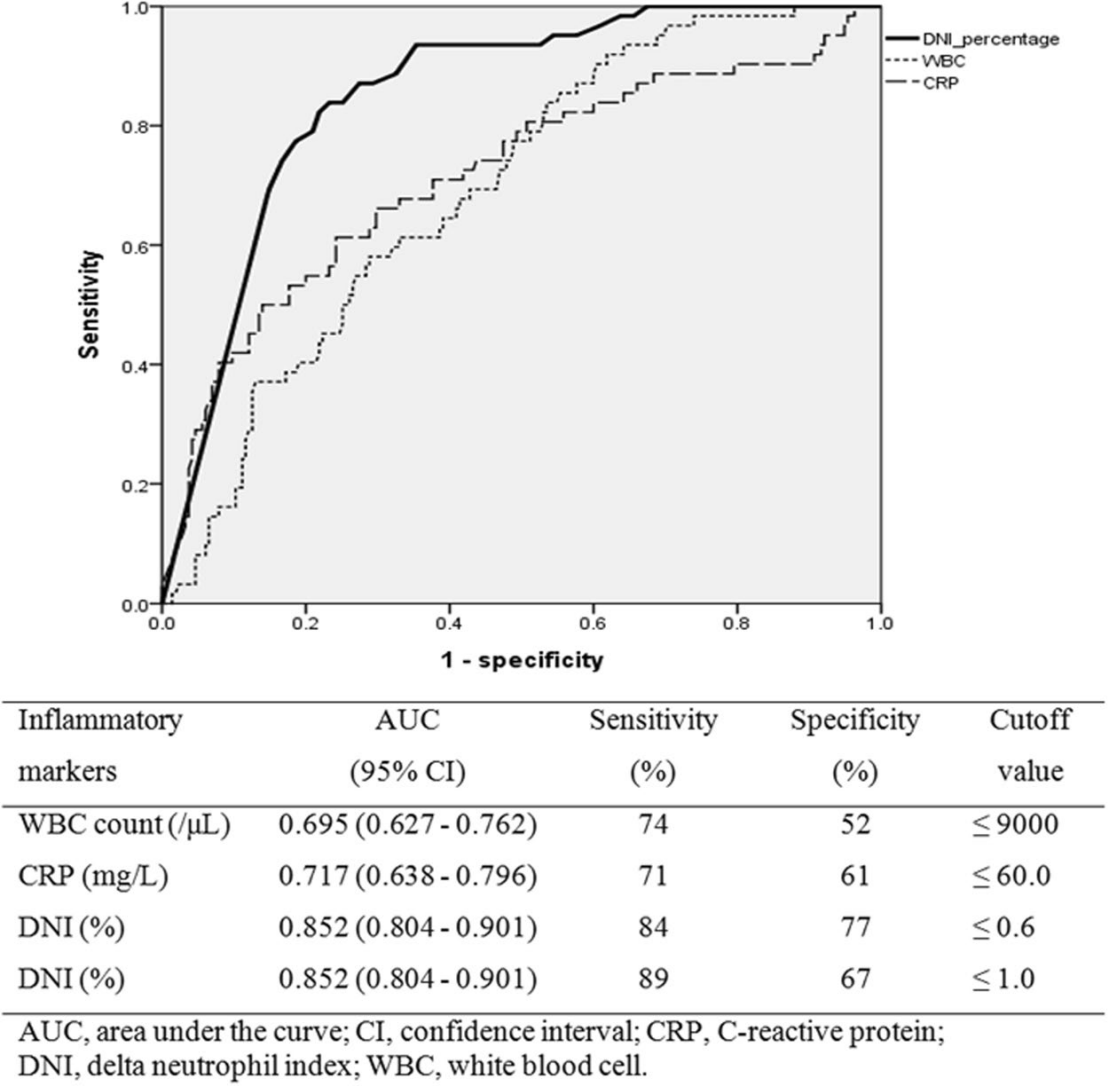
Receiver operating characteristic curve analysis of inflammatory markers for diagnosing pulmonary tuberculosis. AUC, area under the curve; CI, confidence interval; CRP, C-reactive protein; DNI, delta neutrophil index; WBC white blood cell.

We performed a ROC curve analysis of inflammatory markers, including WBC, the DNI, and CRP for diagnosing PTB (Fig. 2). The discriminatory power of the DNI for diagnosing PTB was high, with 84% sensitivity and 77% specificity at a DNI cut-off ≤ 0.6% (AUC, 0.852; 95% confidence interval [CI] 0.804–0.901) and 89% sensitivity and 67% specificity at a DNI cut-off ≤ 1.0% (AUC, 0.852; 95% CI 0.804–0.901), which were higher than those of WBC or CRP. The overall diagnostic sensitivity, specificity, PPV, and NPV for PTB were 84% (52/62), 77% (165/215), 51% (52/102), and 94% (165/175) at a DNI cut-off ≤ 0.6%, respectively, and were 89% (55/62), 67% (145/215), 44% (55/125), and 95% (145/152) at a DNI cut-off ≤ 1.0%, respectively.

### Comparisons of initial chest radiographic findings between patients with PTB and CAP

Initial chest radiography images were available for all patients, and the dominant pattern and main lobes involved were different between patients with PTB and CAP (Table 3). Most patients with PTB (75%, n = 46) had nodules with or without linear opacities, and more than half (58%, n = 36) of patients had only upper lobe involvement. In contrast, 55% (n = 120) of patients with CAP had consolidation, 20% (n = 42) had consolidation with ground glass opacity, and most patients (86%, n = 185) had lower lobe involvement with or without upper lobe involvement.

**Table 3:** Comparisons of initial chest radiographic findings between patients with PTB and CAP.

Characteristics	PTB (n = 62)	CAP (n = 215)	*P*-value
Dominant pattern			<0.001
Consolidation + GGO	—	42 (20)	
Consolidation + nodules	2 (3)	32 (15)	
Consolidation	2 (3)	120 (55)	
GGO	—	15 (7)	
Nodules ± linear opacities	46 (75)	6 (3)	
Cavity	10 (16)	—	
Effusion	2 (3)	—	
Mainly involved lobe			<0.001
Upper lobes only	36 (58)	30 (14)	
Lower lobes ± upper lobes	26 (42)	185 (86)	
Bilateral	17 (27)	71 (33)	0.442

Data are shown as number (%). *CAP* community acquired pneumonia; *GGO* ground glass opacity; *PTB* pulmonary tuberculosis; *–* negative.

### Factors associated with predicting PTB

We also performed univariate and multivariate analyses with a logistic regression model to determine the factors associated with predicting PTB (Table 4). The variables used were ex- or current smoker (n = 86), no fever (n = 152), upper lobe involvement only (n = 66), and DNI ≤ 1.0% (n = 125). No fever (odds ratio [OR], 6.282; *P* < 0.001), upper lobe involvement only (OR, 6.784; *P* < 0.001), and DNI ≤ 1.0% (OR, 15.265; *P* < 0.001) were significantly associated with predicting PTB.

**Table 4:** Univariate and multivariable analysis with logistic regression model for factors predicting PTB.

Variable	Univariate analysis		Multivariable analysis	
	OR (95% CI)	*P*-value	Adjusted OR (95% CI)	*P*-value
Ex- or current smoker (n = 86)	1.559 (0.864–2.812)	0.140	2.088 (0.922–4.728)	0.077
No fever (n = 152)	8.059 (3.659–17.748)	<0.001	6.282 (2.545–15.507)	<0.001
Upper lobe involvement only (n = 66)	8.538 (4.525–16.113)	<0.001	6.784 (3.031–15.182)	<0.001
DNI ≤ 1.0 (%) (n = 125)	16.276 (7.050–37.574)	<0.001	15.265 (6.030–38.654)	<0.001

*CI* confidence interval; *DNI* delta neutrophil index; *OR* odds ratio; *PTB* pulmonary tuberculosis.

## Discussion

The most important finding of our study was that initial DNI values were significantly lower in patients with PTB compared with patients with CAP, and the discriminatory power of the DNI for diagnosing PTB was higher than commonly used inflammatory blood markers, such as WBC or CRP, suggesting the utility of the DNI to discriminate PTB from CAP. Moreover, given that the relatively high sensitivity (89%) and NPV (95%) for diagnosing PTB at a DNI cut-off ≤ 1.0%, the initial DNI value may be useful to rule out the possibility of PTB when suspected patients have DNI values > 1.0%.

Identifying PTB is a troublesome clinical problem in tuberculosis-endemic areas, because failure to discriminate PTB from CAP may result in a considerable public health and economic burden; thus, efforts have been made to distinguish PTB from other respiratory diseases. For example, Liam *et al*. reported clinical features of 17 patients with PTB that distinguished them from 329 patients with CAP, and showed that a longer duration of symptoms >2 weeks, night sweats, upper lobe involvement, cavity on radiography, and lower WBC count were predictive of PTB^6^. Ugajin *et al*. reported that serum procalcitonin was significantly lower in patients with PTB than in patients with CAP; their study included 102 patients with PTB, 62 patients with CAP, and 34 healthy controls^11^. Kang *et al*. also found that both serum procalcitonin and CRP levels were lower in 30 patients with PTB than in 57 patients with CAP^10^. Recently, a new biomarker such as triggering receptor expressed on myeloid cells (TREM) has been evaluated in sputum of patients with PTB and CAP to discriminate these diseases. The TREM is a novel activating receptors of the Ig superfamily expressed on human myeloid cells, and its expression is upregulated by extracellular bacteria and fungi but is weak in mycobacterial, viral, intracellular bacterial, and noninfectious inflammatory disorders^24,25^. The soluble form of the receptor, s-TREM-1, can be measured in plasma and other body fluids such as pleural or peritoneal fluid and may differentiate infectious from non-infectious causes. However, the protein was upregulated in sputum among both patients with CAP and PTB, and did not help to differentiate between the two diseases^12^. To date, sufficient data are not available on accurate and easily applicable markers, and no data exist on utility of the DNI value. Given that the DNI is a convenient test that can provide reliable data rapidly in routine blood tests and considerable discriminating power, even after adjusting for other clinical parameters in a multivariate analysis, the DNI may have an important supplementary role in diagnostic exclusion of PTB from CAP.

Our results are partly explained by the observation that tuberculosis is a relatively chronic inflammatory process associated with a delay in onset of adaptive immunity and granuloma formation, after a series of complex interactions with the host and mycobacteria^1,2^. Thus, the proportions of immature granulocytes could be relatively lower in mycobacterial infection than in bacterial or viral infections such as CAP. Actually, three of the 10 PTB patients with bacterial co-infections had relatively higher DNI levels (≥1.0%) in our study, which supports this hypothesis. Similar to our findings, recent studies that validated the DNI in various clinical scenarios have also demonstrated different DNI levels between bacterial and viral infections or in infectious pyelonephritis versus organ rejection. However, there is a lack of data regarding DNI levels among non-tuberculosis patients with PTB and CAP. In these contexts, our findings could have clinical significance, and although the inflammatory response remains speculative, may partly help to further understanding of the pathophysiology of PTB infection.

Our study had several limitations. First, we included only patients with PTB or CAP and compared the two groups with a retrospective study design. Thus, further prospective studies including various respiratory diseases are needed to accurately evaluate clinical applicability of the DNI for discriminating PTB. Second, we excluded severe cases requiring intensive care unit admission due to severe respiratory failure or shock. However, this was because most cases admitted to the intensive care unit were CAP with extremely high DNI values to up 28% and only a few cases of PTB had clinical instability in actual clinical practice, so we excluded the severe cases. Finally, the study population was relatively small, and the study was conducted at a single center with relatively short study period. Thus, we believe that our findings should be further testified in a validation set. In conclusion, we showed that initial DNI values were lower in patients with PTB than in those with CAP, and that an initially elevated DNI value (>1.0%) may be useful to rule out the possibility of PTB due to its high NPV.
